# Economic Valuation of Balkan Chamois Conservation

**DOI:** 10.3390/ani13040691

**Published:** 2023-02-16

**Authors:** Vasilios Liordos, Maria Antoniadou, Vasileios J. Kontsiotis

**Affiliations:** Department of Forest and Natural Environment Sciences, International Hellenic University, P.O. Box 172, 66100 Drama, Greece

**Keywords:** caprinae, stated preference, random utility, cognitions, theoretical construct, econometric model

## Abstract

**Simple Summary:**

The Balkan chamois is an agile mammal of the rocky mountain slopes of the Balkan peninsula. In Greece, its southernmost limit, it exists in six population groups. Illegal hunting and the isolation of populations are the major threats to the species. Therefore, conservation actions, such as the control of illegal hunting and the creation of wildlife corridors are required for securing viable populations. We used an econometric model to estimate the willingness to pay (WTP) for chamois conservation. People from the region of Eastern Macedonia and Thrace participated in our survey. We asked them if they were WTP an annual tax for the next five years for implementing conservation actions for the chamois. Most of the surveyed people stated a considerable WTP that could yield adequate resources for funding relevant conservation actions. We also examined the effects of several factors on WTP. We found that increasing the knowledge about and improving the attitudes toward the species, targeting groups, such as males, those less educated, and who have not seen the species in the wild, through suitable education and outreach programs would increase public support and WTP for the species. Our findings would help successfully implement conservation plans for the chamois.

**Abstract:**

The Balkan chamois (*Rupicapra rupicapra balcanica*) is a caprine of the rocky mountain slopes, threatened in Greece by illegal hunting and population isolation. We used a contingent valuation method to assess the willingness to pay (WTP) for chamois conservation of 500 residents of the region of Eastern Macedonia and Thrace. Most of the participants (61.6%) were WTP a mean of EUR 41.6 for chamois conservation, totaling EUR 6.03 million for the target population. Attitudes toward and knowledge about chamois, moralistic worldviews (spiritual reverence and ethical concern for nature and wildlife), participation in wildlife-related consumptive outdoor activities (i.e., hunting and fishing), intention to participate in conservation actions for the species, and encounters with the species in the wild were positively associated with WTP for its conservation. Dominionistic worldviews (humans have mastery, physical control, and dominance of wildlife) were negatively associated with WTP, while highly educated females with high income were more WTP for implementing relevant conservation actions. Factors involving previous knowledge of the chamois positively influenced the WTP, thus, confirming the construct’s bias toward charismatic species. The findings show that Greek residents highly value the chamois and its conservation and would be useful for advising this process and achieving its conservation management.

## 1. Introduction

The Balkan chamois (*Rupicapra rupicapra balcanica*), hereafter chamois, is the southernmost subspecies of the northern chamois *R. rupicapra*. It occurs in nine Balkan countries, and its population has been estimated at 9100–10,285 individuals [1]. In Greece, the southern limit of chamois distribution, the species is fragmented into 30 subpopulations, due to both the natural isolation of its favorite mountaintop habitat and anthropogenic land conversion for agriculture and housing, which form 6 main population groups [1,2]. The Greek population of the chamois has been estimated at 1180–1765 individuals [1,3]. This population has more than doubled during the last two decades because of conservation actions, especially the hunting ban [2,3,4]. Despite this population increase, the chamois is strictly protected by European and Greek law, assessed as inadequate–bad conservation status and listed in the Annexes II and IV of the 92/43/EEC Habitat Directive [5], classified as near threatened in the Red Data Book of Threatened Vertebrates of Greece [6], and its hunting has been banned since 1969. Isolation due to fragmentation and illegal hunting are the major current threats to Greek chamois populations [2,4]. The remarkable genetic variability found in the Greek populations of the chamois emphasizes the urgent need for conservation actions focusing on preventing further fragmentation and controlling illegal hunting to maintain viable populations [4]. Public support and fund acquisition are among the most critical factors for successfully implementing wildlife conservation programs [7,8]. The public is also the main source of revenue for both governments and non-governmental organizations. Therefore, knowing the public attitudes toward chamois and the willingness to support and fund relevant conservation programs are necessary for informing successful chamois conservation management.

Wildlife conservation cannot be directly valued, as there is no market where it can be traded. Economists have used the contingent valuation method (CVM), a practical approach for valuing non-market goods using questionnaire surveys to determine the willingness to pay (WTP) by creating a hypothetical market [9,10]. The CVM is a useful approach for estimating the availability of public funds for wildlife conservation [9]. It can also be an index of public support for the conservation of a species and, more importantly, the proposed funding and conservation scheme. Furthermore, the effects of cognitive and environmental factors on WTP can be assessed and differences among stakeholder groups identified. The CVM has been used for determining the WTP for the conservation of many wildlife species because of the wealth of information it can provide (e.g., [11,12,13,14,15,16,17,18,19,20,21,22,23]). The CVM has been previously used in Greece for determining the WTP for bat conservation [24].

People with positive attitudes toward wildlife are more likely to support species conservation than people with negative attitudes [7,25,26,27]. People’s valuations of wildlife species often depend on their knowledge or information about these species [28]. Knowledge of the existence and about the ecology, biology, and habits of wildlife species is usually associated with positive attitudes toward them [29]. Previous studies have reported that high knowledge about certain species corresponded to high support for their conservation and management [7,25]. Moralistic and dominionistic worldviews have proved important predictors of the support and acceptance of wildlife conservation and management strategies. Moralistic worldviews refer to the respect and ethical treatment of nature and wildlife. In contrast, dominionistic worldviews refer to the control, use, and domination of humans over wildlife. Human dimensions research commonly reports that moralistic worldviews are associated with higher support of wildlife conservation than dominionistic worldviews [7,30,31,32,33].

Wildlife-related outdoor recreational activities can be classified as consumptive, involving the handling and killing of animals and including hunting and fishing, and non-consumptive, involving the observation of animals from a distance without harm and including wildlife watching and photography. Both consumptive and non-consumptive recreationists are more involved in conservation activities than non-recreationists [34]. Additionally, consumptive recreationists, such as hunters, are known to participate in wildlife conservation actions for both game and non-game species [35,36]. Age, gender, income, and level of education are among the sociodemographic characteristics most often included in models for predicting the support and WTP for wildlife species conservation. Young, educated females with high income are usually more supportive and WTP for the conservation of wildlife species [7,24,30,37].

This study’s aims were to (a) estimate the WTP for chamois conservation of Greek residents using the CVM and (b) assess the effects of attitudes toward and knowledge about chamois, worldviews, participation in conservation actions and wildlife-related outdoor activities, and sociodemographic characteristics (age, gender, income, and educational level) on WTP.

## 2. Materials and Methods

This study was conducted according to the guidelines of the Declaration of Helsinki and adhered to the ethical standards laid out by the Research Ethics and Deontology Committee of the International Hellenic University. We de-identified questionnaires, sought informed consent from all the participants, and maintained anonymity at all stages of this research. As part of their review, the Research Ethics and Deontology Committee of the International Hellenic University has determined that this study is no more than minimal risk and exempt from ongoing institutional review oversight (REDC-IHU-25619.19).

### 2.1. Sample Collection

This study was carried out in the region of Eastern Macedonia and Thrace, Northern Greece (40°06′04″ Ν, 20°40′56″ E–41°024′16″ Ν, 23°40′27″ E), with a population of about 608,000 people in 235,349 households [38]. One of the six main Greek chamois groups, the Rhodope Mountain range population, lives in the area, currently consisting of about 260 individuals (41°29′19″ Ν, 24°30′18″ E; [1,3]). We used face-to-face surveys to collect data on WTP for chamois conservation. A sample of 30 residents, randomly selected, was used to assess the clarity of the questions and the required time for the completion of the questionnaire. Then, we surveyed people in most neighborhoods in villages, towns, and cities in the study area. We did so to ensure the representation of residents of different socioeconomic statuses in the survey. Our visits were timed so as to coincide with open market hours when people are more active (9.00–15.00 and 17.00–21.00, from Monday to Saturday). Every fifth person passing in front of the researcher (M.A.) was asked to participate in the survey. Upon acceptance, the participant completed the questionnaire by responding to the questions (respondent-completed survey; [39]). The average questionnaire completion time was estimated at 40 min.

### 2.2. Questionnaire Development

In the first part of the questionnaire, the participants were asked about their willingness to pay for the conservation of the chamois. WTP was estimated in two steps. In the first step, participants were asked: “The chamois population of the Rhodope Mountain range needs protection for its survival. Would you support a governmental management program for the conservation of chamois through the payment of an annual tax for a period of five years?” Two reply options were offered: “yes” and “no”. Then, the participants who answered yes in the first step were included in the second step. The payment card format included nine amounts, as suggested by the literature [40,41]: EUR 1, EUR 5, EUR 10, EUR 20, EUR 40, EUR 80, EUR 150, EUR 300, and EUR 500. Participants were asked to choose how certain or uncertain they were about the payment of each listed amount: “definitely yes”, “probably yes”, “not sure”, “probably no”, or “definitely no”.

In the second part, participants were asked a series of questions and statements concerning sociodemographics and their attitudes toward and knowledge about chamois, their intention to participate in chamois conservation actions, their worldviews, and frequency of participation in consumptive and non-consumptive outdoor activities. Firstly, several sociodemographic characteristics were recorded, such as female or male gender, years of age, higher or lower level of education, and annual income per participant’s household. Secondly, the participants’ attitudes toward, knowledge about, and participation in conservation actions for the chamois and worldviews (adopting the six-item short version of the New Ecological Paradigm [42]) were assessed through six, six and five, and six statements, respectively, on a 5-point scale as: “strongly disagree” (1), “disagree” (2), “neither” (3), “agree” (4), or “strongly agree” (5). Lastly, participants were asked how often they participated in consumptive (i.e., hunting or fishing) and non-consumptive (i.e., bird-watching, nature photography) wildlife-related recreational activities, with possible answers being: “never” (1), “rarely” (2), “sometimes” (3), “often” (4), or “very often” (5).

### 2.3. The Econometric Model

We used a two-step random utility econometric model. The first step involved a simple choice model (yes/no) on the probability of paying for a chamois conservation program. It is a binary logistic model [10] with the WTP (yes = 1, no = 0) as the dependent variable and attitude, knowledge, conservation actions, worldviews, consumptive and non-consumptive recreation, and sociodemographics as the independent variables.

Participants who answered yes in the first model were retained in the second model. We implemented the Welsh–Poe interval model for the analysis of multiple-bounded payment card format data [43]. In particular, we used the “probably yes” model (“definitely yes” and “probably yes” were recoded to “yes”, and “not sure”, “probably no”, and “definitely no” were recoded to “no”) because it gives results similar to other commonly used models, such as dichotomous choice, payment card, and open-ended [41,43]. After the recoding, data could be used as double-bounded [44]. If *A^L^* is the highest “yes” bid that the participants accept and *A^U^* the lowest “no” bid that the participants do not accept, the maximum WTP is *A^L^ ≤ WTP < A^U^* [43], and, given a distribution function *F* for WTP, the likelihood is, as proposed by [43] and validated by field studies, e.g., [24,40,41]:

(1) $$lnL=\sum_{i=1}^{N}[\ln(F(A^{U})-F\left(A^{L}\right)]$$

Additionally, assuming a log-logistic distribution:

(2) $$F\left(A^{U}\right)={\left(1+e^{\delta Χ-\alpha \ln\left(A_{i}^{U}\right)}\right)}^{-1}$$

and

(3) $$F\left(A^{L}\right)={\left(1+e^{\delta Χ-\alpha \ln\left(A_{i}^{L}\right)}\right)}^{-1}$$

where *Χ* is the vector of covariates, and *δ* is the corresponding parameter vector. The parameter *α* corresponds to the bid and can be interpreted as the marginal utility of money. Mean WTP is then calculated as:

(4) $$ΜWTP=e^{\frac{\delta Χ}{\alpha }+{\left(\frac{\alpha^{-1}}{2}\right)}^{2}}$$

### 2.4. Data Analysis

We performed the variance inflation factor with the function vifstep of the usdm R package [45] (VIF < 5) and Spearman correlation with the function cor.test of the ggpubr R package [46] (r_s_ < 0.7) for assessing multicollinearity. We retained all variables in the models because all VIFs were <1.7 and r_s_ < 0.545.

Factor analysis (principal components, varimax rotation) was performed to group knowledge, attitude, and conservation actions statements into important factors. Only factors with eigenvalue ≥1 were retained. Confirmatory factor analysis was used to confirm that worldview statements followed the moralistic–dominionistic theoretical construct. Factor reliability was assessed with Cronbach’s alpha, with α > 0.7 being generally accepted [47].

The first model was fitted with binary logistic regression with binomial distribution and logit link function with the function glm of the stats R package [48]. The logitor and logitmfx functions of the mfx R package [49] were used to calculate odds ratios and marginal effects, respectively. The second interval model was fitted with a log-logistic distribution using the function dbchoice of the DCchoice R package [50], which allows for the implementation of the Welsh–Poe approach [43,44]. The nonparametric bootstrap *bootCI* function was used to estimate confidence intervals (95% CI) for the mean WTP.

Principal component factor analysis was performed with SPSS Statistics and confirmatory factor analysis with SPSS Amos statistical software (version 21.0, IBM Corp., 2012). The binary logistic and interval regression models were performed with R 4.0.2 [48]. The significance level was set at *α* = 0.05.

## 3. Results

### 3.1. Sociodemographics

A total of 500 fully completed questionnaires were collected from 574 residents (87% response rate). The study area’s population ratio was a 50.7% female/49.3% male gender ratio, the age ratios were 29.9%, 34.7%, and 35.4% in the 18–34-, 35–54-, and 55+-year-old age classes, respectively, and the higher/lower educational ratios were 19.2%/80.8% [38]. The sample’s gender (*χ*^2^ = 0.006, *df* = 1, *p* = 0.904), age (*χ*^2^ = 2.812, *df* = 2, *p* = 0.245), and educational level (*χ*^2^ = 0.004, df = 1, *p* = 0.907) structures (Table 1) were not different to that of the population’s.

**Table 1:** Variables used in the willingness to pay (WTP) analysis for the conservation of Balkan chamois.

Variable	Definition	Mean	SD	Min	Max
WTP	A total of 1 if the participant is willing to pay for the conservation of Balkan chamois in Greece.	0.62	0.49	0	1
Attitudes toward Balkan chamois	Attitude factor from exploratory factor analysis in Table 2.	3.30	0.73	1	5
Knowledge about Balkan chamois	Attitude factor from exploratory factor analysis in Table 3.	3.25	1.00	1	5
Participation in actions for the conservation of the Balkan chamois	Conservation actions factor from exploratory factor analysis in Table 4.	3.16	1.10	1	5
Moralistic	Worldview dimension from confirmatory factor analysis in Table 5.	4.79	0.49	1	5
Dominionistic	Worldview dimension from confirmatory factor analysis in Table 5.	2.05	1.29	1	5
Seen Balkan chamois	A total of 1 if the participant has seen Balkan chamois in the wild; 0 if the participant has not seen Balkan chamois in the wild.	0.33	0.47	0	1
Age	Years of age.	43.84	17.86	18	90
Gender	A total of 1 if the participant is a woman.	0.51	0.50	0	1
Level of education	A total of 0 if lower, 1 if higher.	0.19	0.45	0	1
Income	Participant’s household income (EUR × 1000).	16.97	15.45	0	130
Consumptive recreation	How often the participant goes for hunting or fishing (1 = never, 2 = rarely, 3 = sometimes, 4 = often, 5 = very often).	1.12	0.61	1	5
Non-consumptive recreation	How often the participant is involved in outdoor activities other than hunting and fishing (1 = never, 2 = rarely, 3 = sometimes, 4 = often, 5 = very often).	2.65	1.12	1	5

**Table 2:** Results of principal components factor analysis of survey participants’ (*n* = 500) attitudes toward Balkan chamois. Descriptive statistics, factor loadings, factor eigenvalues, % variance explained, and factor reliability are given.

Statements	Mean ^a^	SD	Attitude
Balkan chamois are important features of my local landscape.	3.640	1.198	0.761
Balkan chamois must exist because they are valuable to nature.	3.424	1.260	0.766
Balkan chamois must exist because they are valuable to people.	3.332	1.168	0.847
I am proud of having Balkan chamois in my area.	3.292	1.199	0.584
I feel relaxed when I watch Balkan chamois.	3.036	1.312	0.643
The observation of Balkan chamois offers me aesthetic pleasure.	3.104	1.313	0.493
Eigenvalue			2.881
% Variance explained			43.539
Cronbach’s alpha			0.721

^a^ Range: 1 (strongly disagree)–5 (strongly agree).

**Table 3:** Results of principal components factor analysis of survey participants’ (*n* = 500) knowledge about Balkan chamois. Descriptive statistics, factor loadings, factor eigenvalues, % variance explained, and factor reliability are given.

Statements	Mean ^a^	SD	Knowledge
Balkan chamois can jump up to 2 m high.	3.332	1.231	0.897
The commercial value of the Balkan chamois skin is high.	3.200	1.242	0.403
The commercial value of the Balkan chamois meat is high.	3.260	1.25	0.899
Balkan chamois shed their horns in autumn ^b^.	2.832	1.204	0.878
There are about 2000 Balkan chamois in Greece ^b^.	2.740	1.151	0.933
There are about 200 Balkan chamois in the Rhodope mountain range ^b^.	2.712	1.167	0.945
Eigenvalue			4.308
% Variance explained			71.799
Cronbach’s alpha			0.908

^a^ Range: 1 (strongly disagree)–5 (strongly agree); ^b^ false statements. Reverse coded.

**Table 4:** Results of principal components factor analysis of survey participants’ (*n* = 500) intention to participate in actions for the conservation of Balkan chamois. Descriptive statistics, factor loadings, factor eigenvalues, % variance explained, and factor reliability are given.

Statements	Mean ^a^	SD	Conservation Actions
I would participate in public education and outreach actions for Balkan chamois.	2.480	1.481	0.569
I would participate in conservation actions for Balkan chamois, such as population census, habitat improvement.	3.284	1.381	0.945
I would vote for laws and regulations for the conservation of Balkan chamois in my area.	3.432	1.334	0.929
I would donate money for the conservation of Balkan chamois in my area.	3.264	1.357	0.951
I would urge friends and relatives to participate in actions for the conservation of Balkan chamois in my area.	3.348	1.321	0.958
Eigenvalue			3.902
% Variance explained			76.075
Cronbach’s alpha			0.856

^a^ Range: 1 (strongly disagree)–5 (strongly agree).

**Table 5:** Reliability and confirmatory factor analysis (CFA) of worldview statements.

Worldview Statements	Mean ^a^	SD	CFA	Reliability Analysis		
			Factor Loadings ^b^	Item Total Correlation	Alpha If Item Deleted	Cronbach’s Alpha
*Moralistic*						0.813
Humans must live in harmony with nature in order to survive.	4.852	0.437	0.851	0.665	0.776	
The balance of nature is very delicate and easily upset.	4.772	0.588	0.887	0.723	0.679	
When humans interfere with nature it often produces disastrous consequences.	4.732	0.668	0.848	0.667	0.765	
*Dominionistic*						0.938
Humans have the right to modify the natural environment to suit their needs.	2.104	1.390	0.914	0.817	0.923	
Humankind was created to rule over the rest of nature.	2.040	1.362	0.958	0.903	0.886	
Plants and animals exist primarily to be used by humans.	2.016	1.338	0.957	0.899	0.890	

^a^ Range: 1 (strongly disagree)–5 (strongly agree); ^b^ all *t* values for standardized factor loadings were significant at *p* < 0.001.

Confirmatory factor analysis provided a good fit for the data (*χ*^2^/df = 3.101, RMSEA = 0.044, NFI = 0.985, CFI = 0.991) and supported the moralistic and dominionistic constructs, with factor loadings ≥0.848 (all statistically significant at *p* < 0.001; Table 5). Additionally, the internal reliability of moralistic (*α* = 0.813) and dominionistic (*α* = 0.938) worldviews was high.

### 3.2. Attitudes, Knowledge, Conservation Actions, Worldviews

Exploratory factor analysis determined the following factors. One attitude factor (eigenvalue, 2.9) with high internal reliability (*α* = 0.721), explaining 43.5% of the variance (mean score, 3.305 ± 0.731 SD; Table 2). The participants’ attitudes toward chamois were slightly positive. One knowledge factor (eigenvalue, 4.3) with high internal reliability (*α* = 0.908) explained 71.9% of the variance (mean score, 3.251 ± 1.001; Table 3). The participants’ knowledge about chamois was average. One conservation actions factor (eigenvalue, 3.9) with high internal reliability (*α* = 0.856) explained 76.1% of the variance (mean score, 3.162 ± 1.096; Table 4). The participants’ intention to participate in conservation actions for the chamois was average.

The attitude, knowledge, conservation actions, and moralistic and dominionistic worldview factors were used as predictors in the econometric models.

### 3.3. Willingness to Pay for Balkan Chamois Conservation

Most of the participants were WTP for the conservation of chamois (61.6%). The proportion of correct predictions for the whole sample was 78.8% for the logistic regression, yes/no, model (Table 6). Positive attitudes toward chamois were associated with high WTP (*p* = 0.035), with the probability of WTP increasing by 11.0% per unit of increase in attitudes. Knowledge about chamois was positively associated with WTP (*p* < 0.001), with the probability of WTP increasing by 14.6% per unit of increase in knowledge. The increasing intention to participate in conservation actions for the chamois increased WTP (*p* = 0.039), with the probability of WTP increasing by 6.5% per unit of increase in the intention to participate in conservation actions. Moralistic worldviews were positively associated with WTP (*p* = 0.023), with the probability of WTP increasing by 7.0% per unit of increase in moralistic worldviews. Those who had seen chamois in the wild were 10.0% more likely to be WTP for chamois conservation than those who had not seen chamois in the wild (*p* < 0.001). A high household income was associated with high WTP (*p* = 0.017), with the probability of WTP increasing by 2.6% per unit of increase in income. Those who participated more in consumptive outdoor activities were 11.8% more likely to be WTP for chamois conservation than those who participated less in consumptive outdoor activities (*p* = 0.007).

The second, highest yes/lowest no bids, model estimated the amount that the participants who replied “yes” in the first model were WTP for the conservation of chamois (Table 7). Participants with more positive attitudes toward chamois (*p* < 0.001), who participated more in chamois conservation actions (*p* < 0.001), had higher moralistic (*p* < 0.001) and lower dominionistic (*p* = 0.012) worldviews, had seen chamois in the wild (*p* = 0.007), and participated more in consumptive activities (*p* = 0.022) were WTP a higher amount for chamois conservation than those with less positive attitudes, less knowledge, who participated less in conservation actions, with lower moralistic and higher dominionistic worldviews, and who participated less in consumptive outdoor activities. Highly educated (*p* = 0.022) females (*p* = 0.009) with higher income (*p* = 0.001) were WTP a higher amount for the conservation of chamois than less educated males with lower income.

The mean amount of WTP was estimated at about EUR 41.6 (95% CI: 33.4–49.9). Considering the mean value, confidence intervals, the proportion of the participants who were willing to pay, and the number of households in the study area, the amount of EUR 6.03 million (min EUR 4.83 million, max EUR 7.23 million) could be collected in taxes for the conservation of chamois populations.

## 4. Discussion

### 4.1. WTP for Balkan Chamois Conservation

A large proportion of the participants were WTP for chamois conservation by paying an annual tax for implementing a five-year conservation plan. Based on the mean annual WTP, a considerable amount could be collected annually. The funds necessary for the conservation of chamois populations are not known but, considering the total amount of money that could be secured, we expect that a new annual tax would be sufficient for implementing conservation plans. Although considerable, a further increase in public support, as expressed by WTP proportions, would be desirable because, in addition to necessary funds, public support is also critical for successful wildlife conservation [7,8].

Illegal hunting is considered a major threat to wild mammals globally, more so to small and isolated populations [51,52]. Illegal hunting has also been recognized as the major threat to chamois in the study area [2,4]. Chamois meat and skin are both valuable in the market. Preliminary investigations have revealed that chamois are predominantly hunted for their skin and that both Greeks and Bulgarians participate in illegal actions [2,4]. However, reliable data on the intensity of illegal hunting and its exact drivers are lacking, and hence, the long-term consequences for the chamois population cannot be assessed. Possible social and economic drivers of illegal hunting include poverty and income generation, demand for wildlife products, recreational needs, trophy acquisition, and a behavioral intention to hunt illegally [53]. Future research should determine both the current and future effects of illegal hunting on chamois populations and its proximate and underlying drivers. In doing so, support for the caprine will increase, and its future will be brighter.

Our results fell within and to the upper part of the 31% to 80% WTP rates and EUR 0.0 to EUR 71.2 mean WTP reported from similar studies [11,12,14,15,19,20,40,54]. This trend did not change after adjusting for inflation and GDP (see Table 4 in Liordos et al. [24]). These findings suggest that there is considerable interest in chamois conservation among the Greek public. Higher WTP values have been generally reported for mammals and birds [11,14,19,20] than for reptiles and amphibians [13,23] but not always [12,15]. The public favors the conservation of endangered mammals and birds in comparison to reptiles and amphibians [7,30]. Previous studies have shown that factors such as phylogenetic resemblance to humans [55,56] and physical size [57] are associated with support for endangered species conservation. Additionally, mammals, birds, and fish have a positive social construction, as opposed to reptiles and amphibians [58]. A mean WTP of EUR 21.7 for bat conservation was estimated in a comparable study in Greece [24]. It seemed that the Greek public valued more chamois than bats since it would allocate to them more than double the amount that would allocate to bats. Chamois are charismatic, medium-to-large-sized mammals, while bats, although mammals themselves, are among the most unlikeable and feared and least supported conservation species [7,30].

### 4.2. Predictors of WTP

Attitudes toward chamois were positively associated with higher proportions and amounts of money of WTP for their conservation. Additionally, an observation of the species in the wild improved the proportions and amounts of WTP. On the other hand, knowledge about chamois’ ecology, biology, and habits was positively associated with WTP proportions among our sample participants but not with monetary WTP. Previous studies that have commonly reported that people with positive attitudes toward and observation of certain species, including mammals such as the brown bear (*Ursus arctos*), red deer (*Cervus elaphus*), pygmy rabbit (*Brachylagus idahoensis*), cougar (*Puma concolor*), tiger (*Panthera tigris*), leopard (*Panthera pardus*), and giant panda (*Ailuropoda melanoleuca*), are associated with higher support and WTP for their conservation [7,19,25,26,27,30]. Other studies have also shown that knowledge about wildlife in general and certain species in particular improved behavior toward and WTP for the conservation of species, such as snakes [25,59], marine turtles [60], and sharks [61]. Knowledge is seen as a prerequisite for someone’s behavior [62], influencing individuals’ valuations of environmental commodities [63]. However, although important, knowledge about biodiversity has been a relatively minor factor in predicting whether members of the public will know about specific pro-environmental behaviors they can take, whether they will actually undertake such behaviors, and whether they will support their conservation [64], as compared with attitudes, charisma, knowledge of a species, and its phylogenetic resemblance to humans [7,30,65,66,67,68]. In line with these findings, attitudes toward and knowledge of chamois’ existence were more important predictors of WTP than knowledge about the caprine’s ecology, biology, and habits in our study. It seemed that being able to recall the image of chamois in someone’s brain functioned as a positive stimulus. The chamois’ figure, posture, and physical size most likely invoked positive emotions and attitudes due to its phylogenetic proximity to humans [65,68].

Moralistic worldviews were positively associated with the proportion and, more so, with the amount of WTP for chamois conservation. Dominionistic worldviews were negatively associated with the amount of WTP. People with moralistic values are fervent champions of animals and nature in general and, therefore, are expected to support actions for their conservation [42]. In contrast, those with dominionistic values are utilitarian and give priority to wildlife use over conservation. Those who hold moralistic worldviews are more supportive of endangered species conservation than those who hold dominionistic worldviews [7,30]. Overall, moralistic worldviews were better predictors of WTP for chamois conservation than dominionistic worldviews. Previous studies have also found that moralistic cognitions are better predictors of wildlife conservation [7,8,30], while dominionistic cognitions are better predictors of wildlife impact management [69,70,71,72]. As expected, survey participants who intended to participate in chamois conservation actions were also more WTP for its conservation. People who participate in wildlife conservation actions have a special interest in wildlife, have or acquire knowledge about wildlife in general and certain species in particular, are champions of animal welfare, and are opposed to wildlife management strategies that can harm wildlife [73].

Participants who were engaged in wildlife-related consumptive activities were more WTP than participants who did not engage in such activities. Consumptive users of wildlife, such as hunters and fishers, are nature enthusiasts that enjoy nature and wildlife and have a special interest in conservation issues, especially those concerning their favorite game, and often engage in conservation actions. [35,36]. Greek hunters had greater knowledge of the existence and about the ecology, biology, and behavior of wildlife species, both game and non-game, than non-hunters [74]. The greater experiential knowledge of consumptive users about wildlife, their interest in wildlife conservation, and the increased possibility to encounter chamois in the wild might explain their greater support and WTP for the conservation of the species.

Our results show that a high amount of WTP was associated with females and a high level of education. High income was associated with both high proportions and a high amount of WTP. In a comparable CVM study in Greece, WTP was associated with a high level of education but not with gender or income [24]. Other similar studies have reported variable findings. Gender, income, and educational level were associated with WTP for gray wolf (*Canis lupus*) and Mexican free-tailed bat (*Tadarida brasiliensis mexicana*) conservation [11,37]. Educational level and income were positively associated with WTP for white stork (*Ciconia Ciconia*) and giant panda conservation. Gender, income, and educational level did not affect the WTP for the conservation of the tiger and the Mauritian flying fox (*Pteropus niger*) [54,75]. As WTP increased with income in our study, it represented a “normal good” for the Greek residents according to economic principles [76].

Attitudes, worldviews, participation in conservation actions, educational level, income, and being female were stronger predictors of the amount than the proportion of WTP. This larger effect on the amount than the proportion of WTP of these factors suggests that conservation initiatives should focus not only on increasing the proportions of those not WTP for chamois conservation, such as those with more negative attitudes, less moralistic and dominionistic worldviews, who do not participate in conservation actions, are male, and have low education and income but also those that were WTP. Given that the second WTP model included only those WTP for chamois conservation, the stronger effect of the above-mentioned factors suggests that there was variation in the amount they were WTP among those with more positive attitudes and moralistic and dominionistic worldviews, who participate in conservation actions, are female, and have high education and income. In doing so, both higher support and larger funds would be secured for the conservation of the species. On the other hand, knowledge, non-consumptive recreational activities, and having seen the species in the wild were better predictors of the amount than the proportion of WTP. This suggests that there was a threshold above which an increase in knowledge, non-consumptive users, and those who have seen the species in the wild did not improve the amount of WTP.

### 4.3. Management Implications

Survey participants stated support and WTP for the conservation of chamois. The collection of necessary funds through an annual tax would be sufficient for effectively applying suitable conservation actions, especially controlling illegal hunting of the chamois population and determining its drivers in the study area [2,4]. Outside the study area, wildlife corridors are necessary for connecting population groups, especially in the Pindus Mountain range. Attitudes toward chamois were neutral and knowledge about the species average. Additionally, our results reveal that attitude was positively associated with the proportion and amount of WTP, while knowledge was positively associated only with the amount of WTP for chamois conservation. These results suggest that there is room for a further increase in attitudes and knowledge, which in turn will increase the support and WTP—a prerequisite for the caprine’s successful conservation [7,8].

Public education and outreach programs should be designed and implemented, aiming at increasing the knowledge of people about chamois distribution and conservation status, especially in the targeted areas, biology, ecology, and behavior. Experiential activities, such as chamois observation and identification of biomarkers would also improve knowledge about and attitudes toward the species [77]. Such programs and activities should be primarily addressed to groups that the results identified as having low support for chamois conservation, such as those with more negative attitudes, less knowledge and moralistic and dominionistic worldviews, who have not seen the species in the wild and do not participate in consumptive activities and conservation actions, are male, and have low education and income. Additionally, the greater effect of several factors on the amount than the proportion of WTP suggests that considerable variation also existed within groups with a higher amount of WTP, such as those with more positive attitudes and moralistic and dominionistic worldviews, who participate in conservation actions, are female, and have high education and income. Such groups should also be the target of education and outreach campaigns. Outreach campaigns should also aim at discussing suitable conservation actions with the public [78]. Actions that are not perceived well by the public might not be acceptable and, thus, jeopardize the conservation outcomes.

### 4.4. Study Limitations

The limitations of our survey methodology should be considered when using the findings from this study. Potential observer bias, bias toward people that are not in a hurry, and giving socially acceptable answers to sensitive questions are the main limitations of face-to-face surveys [39]. Surveys were carried out by one researcher to deal with interobserver bias. We kept the surveys anonymous and opted for the respondent-completed approach [39] to avoid eliciting socially desirable answers. Additionally, the high response rate to our survey suggests that the effect of people’s time availability on their decision to participate in the survey was minimal. However, future research should further investigate the potential differences between face-to-face and other methods, such as online, mail, and workplace surveys.

## 5. Conclusions

The Greek public showed support and assigned a considerable monetary WTP for chamois conservation. These findings suggest that a considerable amount of money could be collected through an annual tax for the conservation of the species. Attitudes toward and knowledge about chamois, moralistic worldviews, participation in wildlife-related consumptive outdoor activities, intentions to participate in conservation actions for the species, and encounters with the species in the wild were positively associated with WTP for its conservation. Dominionistic worldviews were negatively associated with WTP, while highly educated females with high income were more WTP for implementing conservation actions for the caprine. Future research should focus on increasing knowledge about and improving attitudes toward chamois through education and outreach programs, especially targeting groups showing low support and amount of WTP. Our findings will be useful for advising this process and achieving the conservation of this charismatic species.

## Figures and Tables

**Table 6:** Results of the binary logistic regression willingness to pay model (yes/no, *n* = 500).

Variable	Odds Ratio	Marginal Effects	*p*
Attitude	1.594	0.110	*0.035*
Knowledge	1.850	0.146	<0.001
Conservation actions	1.314	0.065	*0.039*
Moralistic	1.343	0.070	*0.023*
Dominionistic	0.929	−0.017	0.368
Seen in the wild	1.523	0.100	*<0.001*
Age	0.992	−0.002	0.461
Gender (female)	1.023	0.005	0.910
Level of education (higher)	0.949	−0.012	0.831
Income	1.117	0.026	*0.017*
Consumptive recreation	1.645	0.118	*0.007*
Non-consumptive recreation	1.148	0.033	0.131
Nagelkerke’s *R*^2^	0.293		
−2LogLik	581.036		
AIC_c_	608.170		

**Table 7:** Results of the log-logistic regression willingness to pay model (highest yes/lowest no bids, *n* = 308).

Variable	Coefficient	SE	*p*
Attitude	0.472	0.122	*<0.001*
Knowledge	0.097	0.082	0.233
Conservation actions	0.262	0.070	*<0.001*
Moralistic	0.515	0.131	*<0.001*
Dominionistic	−0.127	0.048	*0.012*
Seen in the wild	0.359	0.134	*0.007*
Age	−0.003	0.007	0.643
Gender (female)	0.165	0.115	*0.009*
Level of education (higher)	0.112	0.157	*0.027*
Income	0.005	0.000	*0.001*
Consumptive recreation	0.215	0.116	*0.022*
Non-consumptive recreation	0.03	0.062	0.627
Nagelkerke’s *R*^2^	0.314		
−2LogLik	602.675		
AIC_c_	628.918		
Mean WTP (EUR)	41.595		
95% CI of mean WTP (EUR)	33.347–49.862		

## Data Availability

The data presented in this study are available on reasonable request from the corresponding author.
